# Antimicrobial resistance of *Streptococcus pneumoniae* from invasive pneumococcal disease in Brazil

**DOI:** 10.62675/2965-2774.20250204

**Published:** 2025-02-28

**Authors:** Pedro Kurtz, Pedro Fernandez Del Peloso, Fernando Augusto Bozza

**Affiliations:** 1 Instituto D’Or de Pesquisa e Ensino Rio de Janeiro RJ Brazil Instituto D’Or de Pesquisa e Ensino - Rio de Janeiro (RJ), Brazil.; 2 Instituto Estadual do Cérebro Paulo Niemeyer Department of Intensive Care Medicine Rio de Janeiro RJ Brazil Department of Intensive Care Medicine, Instituto Estadual do Cérebro Paulo Niemeyer - Rio de Janeiro (RJ), Brazil.; 3 Richet Laboratory Rio de Janeiro RJ Brazil Richet Laboratory - Rio de Janeiro (RJ), Brazil.; 4 Fundação Oswaldo Cruz Instituto Nacional de Infectologia Evandro Chagas Rio de Janeiro RJ Brazil Instituto Nacional de Infectologia Evandro Chagas, Fundação Oswaldo Cruz - Rio de Janeiro (RJ), Brazil.

Invasive pneumococcal infections, which are predominantly caused by *Streptococcus pneumoniae* *(S. pneumoniae),* remain a significant health concern throughout the world. *S. pneumoniae* is the principal pathogen in community-acquired pneumonia and bacterial meningitis, which often lead to severe complications; moreover, these diseases require intensive care and are associated with increased mortality rates. A particularly concerning trend involves the emergence of penicillin- and cephalosporin-nonsusceptible pneumococcal strains, which pose a growing challenge to public health worldwide.^(1–4)^

Our study aimed to assess the prevalence of antimicrobial-resistant pneumococci in the hospital setting in Rio de Janeiro, Brazil. Between January 1^st^, 2021, and December 31^st^, 2023, we analyzed all 411 consecutive isolates of *S. pneumoniae* from blood cultures of individual patients from 16 tertiary hospitals. These bacterial isolates were identified utilizing MALDI-TOF mass spectrometry, and antibiotic susceptibility testing was conducted with the VITEK 2 XL system using the AST-ST03 card. We conducted parallel resistance screening for beta-lactams using oxacillin discs and determined the minimum inhibitory concentrations (MICs) for penicillin and ceftriaxone, whereby we employed concentration gradient strips for subsequent comparative analysis. The interpretation of the results adhered to the standards of EUCAST version 14.0.^(5)^

**Figure 1:**
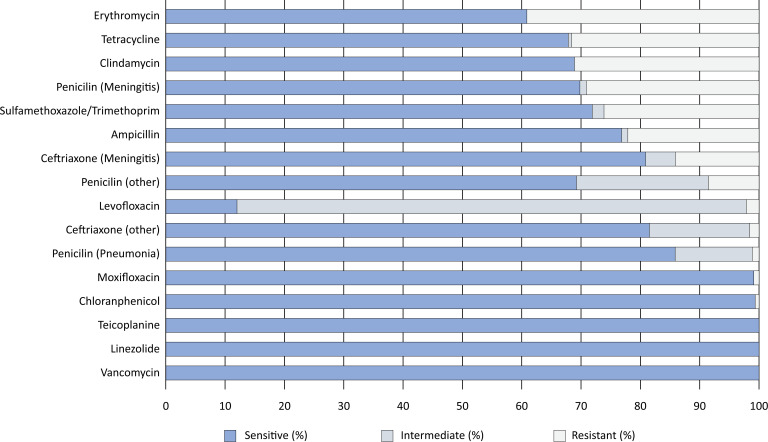
Antimicrobial susceptibility of *Streptococcus pneumoniae*.

The median age of the patients was 46 years (interquartile range 4 - 71), and 48% were female, with 141 (34%) children and 136 (33%) elderly patients being analyzed (Table 1). When MIC breakpoints for meningitis treatment were considered, we detected in vitro antimicrobial nonsusceptibility (resistance) in 14% of the isolates for ceftriaxone and 29% for penicillin. When the MIC breakpoints for other infections were considered, resistance was present in 2% of the isolates for ceftriaxone, 8% for penicillin, 22% for ampicillin, 26% for sulfatrimethoprim, 1% for moxifloxacin, and 2% for levofloxacin. Remarkably, no resistance was observed for linezolid, teicoplanin, or vancomycin (Figure 1).

**Table 1:** Patient and microbe characteristics

Characteristic		N	n (%)
Age (years)		411	
	< 18		141 (34)
	18 - 40		41 (10)
	40 - 65		(23)
	> 65		136 (33)
Female		411	197 (48)
*S. pneumoniae resistant* to:			
	Penicillin (other infections)	393	31 (8)
	Penicillin (meningitis)	361	105 (29)
	Ampicillin	365	80 (22)
	Ceftriaxone (other)	405	6 (2)
	Ceftriaxone (meningitis)	384	54 (14)
	Erythromycin	373	145 (39)
	Levofloxacin	408	8 (2)
	Sulfamethoxazole/trimethoprim	372	97 (26)
	Moxifloxacin	372	3 (1)
	Teicoplanin	373	0
	Linezolid	376	0
	Vancomycin	402	0

Bars represent the frequency as a percentage. Green, susceptible; yellow, intermediate/susceptible to increased exposure; red, resistant.

Our findings revealed that approximately one in every seven *S. pneumoniae* isolates were nonsusceptible to ceftriaxone as a treatment for meningitis, which remains the first-line therapy recommended by the Brazilian Ministry of Health.^(6)^ Data on ceftriaxone resistance in meningitis vary widely in the literature, with resistances ranging from as low as 1% to as high as 20%.^(1–4)^ Additionally, 17% of isolates had intermediate MICs for ceftriaxone for other infections, such as pneumonia, which would require higher antimicrobial doses or alternate treatments compared to current therapeutic recommendations.^(7,8)^ These results urge a reconsideration of empirical antibiotic strategies for meningitis and possibly for pneumonia. Empirical treatments for community-acquired meningitis should always involve the combination of ceftriaxone and vancomycin, as recommended by international guidelines.^(9–11)^ Our study has evident limitations. First, our data had no information on the serotype distribution among the samples, which has been shown to affect the severity of disease, resistance patterns, and vaccine effectiveness. Nonetheless, serotypes are not usually clinically evaluated or utilized for individual decisions on empirical antibiotic treatment. Second, our database did not include clinical data on the etiology of invasive pneumococcal disease, the utilized antibiotics, or patient outcomes. This clearly limits our conclusions to the microbiological patterns of resistance. Future studies are needed to better understand the impact of these resistance patterns on the antibiotic response and clinical outcomes.
